# Identification and expression profiling of miRNAs in two color variants of carrot (*Daucus carota* L.) using deep sequencing

**DOI:** 10.1371/journal.pone.0212746

**Published:** 2019-03-07

**Authors:** Bhavana Bhan, Archana Koul, Deepak Sharma, Malik Muzafar Manzoor, Sanjana Kaul, Suphla Gupta, Manoj K. Dhar

**Affiliations:** 1 Genome Research Laboratory, School of Biotechnology, University of Jammu, Jammu, India; 2 Plant Biotechnology Division, CSIR—Indian Institute of Integrative Medicine, Jammu, India; National Institute of Plant Genome Research, INDIA

## Abstract

microRNAs represent small endogenous RNAs which are known to play a crucial role in various plant metabolic processes. Carrot being an important vegetable crop, represents one of the richest sources of carotenoids and anthocyanins. Most of the studies on microRNAs have been conducted in the aerial parts of the plants. However, carrot has the rare distinction of storing these compounds in roots. Therefore, carrot represents a good model system to unveil the regulatory roles of miRNAs in the underground edible part of the plant. For the first time, we report the genome wide identification and expression profiling of miRNAs in two contrasting color variants of carrot namely Orange Red and Purple Black using RNA-seq. Illumina sequencing resulted in the generation of 25.5M and 18.9M reads in Orange Red and Purple Black libraries, respectively. In total, 144 and 98 (read count >10), conserved microRNAs and 36 and 66 novel microRNAs were identified in Orange Red and Purple Black, respectively. Functional categorization and differential gene expression revealed the presence of several miRNA genes targeting various secondary metabolic pathways including carotenoid and anthocyanin biosynthetic pathways in the two libraries. 11 known and 2 novel microRNAs were further validated using Stem-Loop PCR and qRT-PCR. Also, target validation was performed for selected miRNA genes using RLM-RACE approach. The present work has laid a foundation towards understanding of various metabolic processes, particularly the color development in carrot. This information can be further employed in targeted gene expression for increasing the carotenoid and anthocyanin content in crop plants.

## Introduction

MicroRNAs (miRNAs) display a class of endogenous and non-coding small RNAs that play regulatory roles in modulating various metabolic processes in both plants and animals [1,2]. They act as *trans*-acting regulators, which modulate the gene expression at the post-transcriptional level and occasionally at the translational level. In plants, these ~21-nucleotide long RNAs are processed from the stem-loop regions of long primary transcripts by a Dicer-like enzyme [3]. Further, they are loaded into a silencing complex known as RNA induced silencing complex (RISC), where they direct the cleavage of complementary mRNAs/targets. The majority of plant miRNA target the encoded transcription factors or other regulatory molecules, such as proteins involved in ubiquitin and RNAi pathways.

miRNAs are crucial regulators of gene expression that affect multiple aspects of plant developmental biology e.g. color development and other metabolic processes [4,5] Mutations occurring during miRNA biogenesis exhibit severe, pleiotropic abnormalities, and plants that over-express/down-regulate particular miRNAs exhibit a wide array of unusual phenotypes. For example, apetala2 (*ap2*) gene is crucial for flower growth and development and represents one of the major targets of miR172. Over-expression of miR172 resulted in the translational inhibition of the *ap2* and *ap2*-like genes and resulted in early flowering and related developmental defects [6].

Both laboratory and computational approaches have revealed the diverse and important roles of miRNAs in plant growth and developmental processes [2,7]. With the advent of small RNA-sequencing technology for profiling the miRNAs in plants, understanding the regulatory roles of miRNAs in various developmental processes like stress, diseases, secondary metabolism, etc. has increased. As a result, the time and cost of sequencing have considerably reduced. Major advantage of the small RNA-sequencing for miRNA profiling includes the precise identification of miRNA sequences and detection of both known and novel miRNAs. Stringent criteria have been established for the annotation of small RNA sequences as miRNAs [3].

The discovery of genes involved in metabolic pathways represents a challenge due to the complexity of interconnected pathways. This becomes all the more difficult as the products generated represent the results of complicated enzymatic processes producing several products, rather than the single compound [8]. The best utility of the deep-sequencing method lies in the identification and differential expression of miRNAs where two contrasting variants are available. Color variants are available in higher plants where they show contrast mainly in different types of colored pigments i.e. carotenoids and anthocyanins in aerial as well as underground parts (reviewed by Dhar et al. [9]). Carrot (*Daucus carota* L.) is one of the richest sources of carotenoids and anthocyanins and has the rare distinction of storing them in roots. Therefore, it represents a good model system to understand the mechanism of carotenoid and anthocyanin biosynthesis and accumulation in roots.

Carrot is an important vegetable grown worldwide and is a biennial in the wild, while the cultivated variants are grown as annuals. Carrot is a diploid species with 2n = 2x = 18. The carrot being a root, is available in varied colors like orange, purple-black, red, white and yellow. The variation in color is from purple-black, caused by the accumulation of anthocyanins, to dark orange, due to the accumulation of carotenoids while red carrot accumulates lycopene and yellow carrots accumulate xanthophylls [10]; white carrot has no carotenoid or anthocyanin accumulation. Iorizzo et al. [11] sequenced high-quality genome of a double-haploid Nantes-type orange carrot and hypothesized that the primary mechanism which regulates the carotenoid accumulation in carrot is not at the biosynthetic level but at the upstream level of photosystem development and functional processes, including photomorphogenesis and root de-etiolation. This indicates that the carotenoid and other pigment pathways are possibly being regulated at the upstream level mainly by the coordinated pairing of miRNAs with the transcription factors (miRNA:TFs) which finally leads to the biosynthesis of different metabolites in the cell [12].

In the present investigation, we report for the first time deep sequencing of miRNAs from two color variants of carrot. Our aim was to identify the miRNAs and their differential gene expression, which might play an important role in color development processes. We also predicted, in-silico characterized and validated the novel miRNAs from both the variants. The carrot miRNA profiling may provide an insight into the molecular mechanisms involved in the development of color as well as other metabolic processes. This will further help in implementation of sustained agricultural production and improved human health.

## Material and methods

### Plant samples

**Fig 1:**
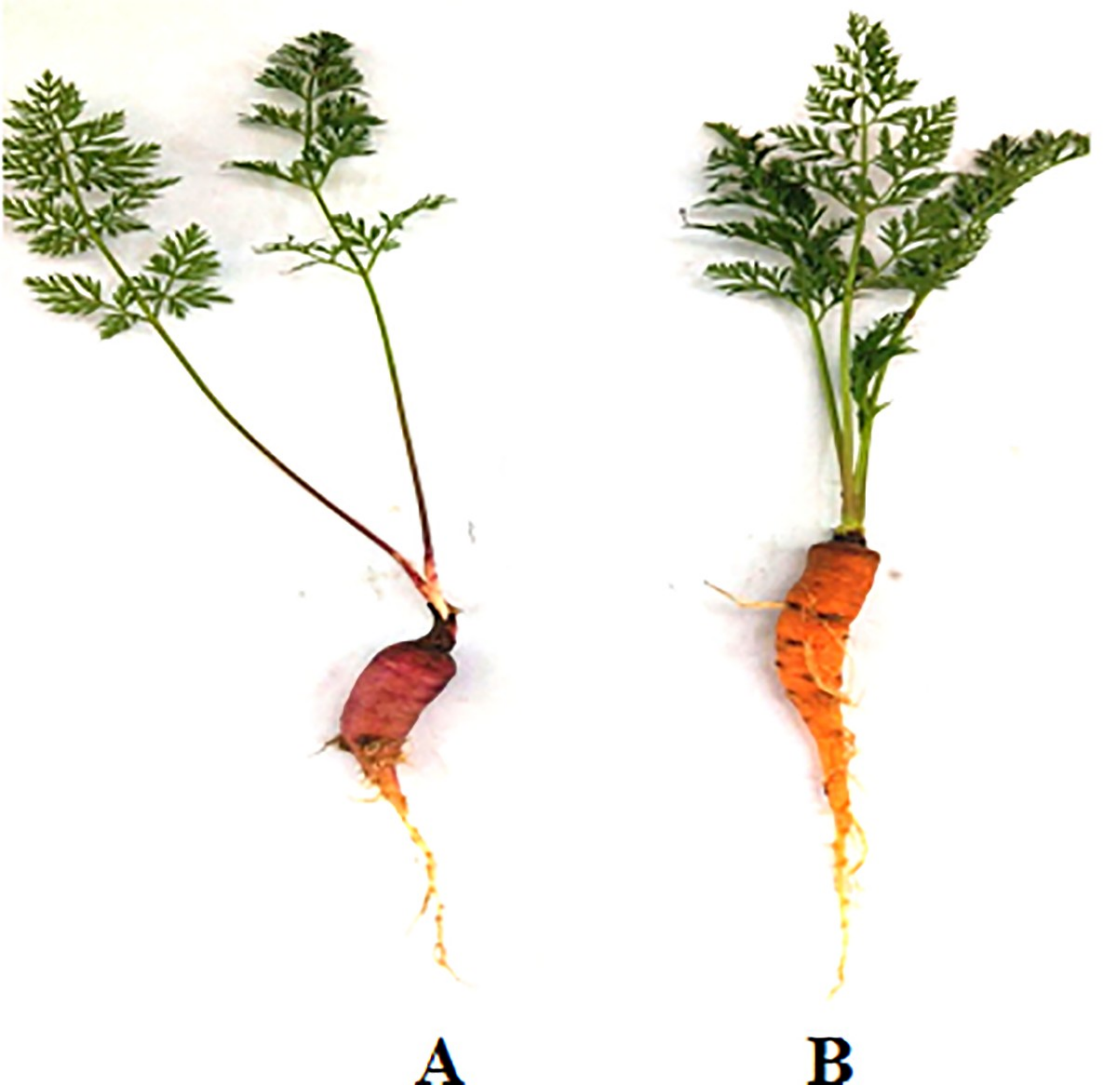
**Carrot variants used during the present study.** A) Purple Black (PB) and B) Orange Red (OR).

We selected two contrasting colored varieties of carrot, Purple Black (PB) and Orange Red (OR) for the deep sequencing of miRNA. PB carrot has dark purple color whereas OR has dark orange color (Fig 1). The seeds of both these varieties were sown during the month of October. Germination of seeds was observed after one week of sowing. The plants were maintained in the Greenhouse at School of Biotechnology, University of Jammu under controlled conditions (16–22°C temperature and 16 hrs photoperiod). Mature roots were harvested after 9 weeks following the date of sowing and stored at -80°C for further use. Purple black and orange red carrots were differentiated on the basis of morphology: PB carrot has dark purple color whereas OR has dark orange color (Fig 1). Different developmental stages were monitored in order to follow the process of carotenogenesis.

### sRNA libraries preparation and sequencing

**Fig 2:**
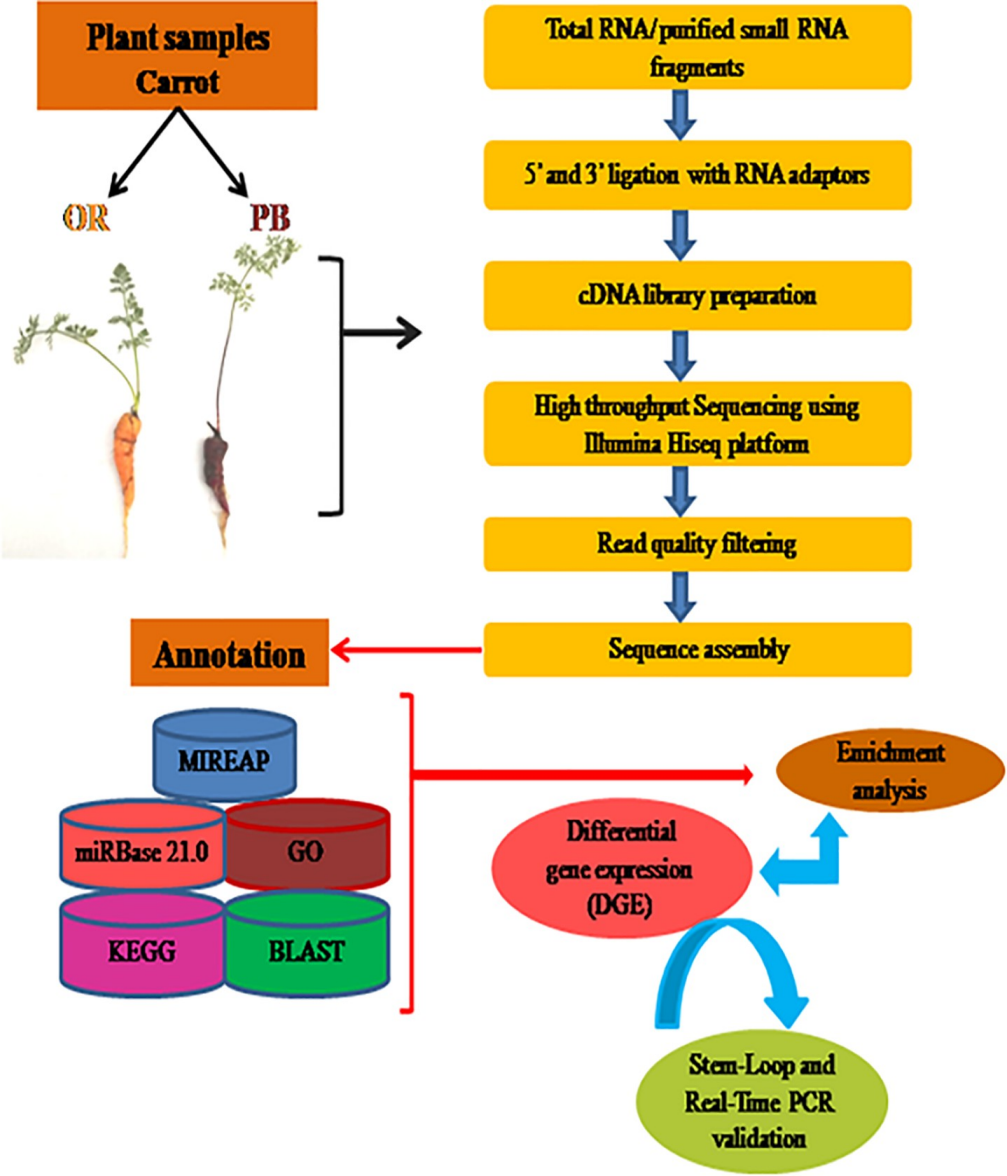
Flow chart of library preparation and sequencing.

Total RNA was extracted from the young roots of both the varieties of carrot (Purple Black and Orange Red) in triplicates using TRIZOL reagent (Invitrogen, USA) according to the manufacturer’s instructions. Both the quality and quantity of the isolated RNA samples were checked using Agilent Bioanalyzer (Agilent Technologies, U.S.A). Further, the RNA samples were run on 15% Tris-Borate-EDTA (TBE) urea denaturating polyacrylamide gel and the 18 to 36 nt small RNA fraction was extracted and eluted. Equal amount of RNA samples, isolated in triplicates from both the varieties, were pooled together and sequenced, respectively. Next, the fractioned sRNA molecules were ligated to 5' and 3' adaptors sequentially and then converted to cDNA by RT-PCR (Fig 2). Size of each library was selected in the range of 145-160bp followed by overnight gel elution and salt precipitation with glycogen, 3M sodium acetate and absolute ethanol. The precipitate was resuspended in nuclease free water. The prepared library was quantified using Qubit Fluorometer and validated for quality by running an aliquot on High Sensitivity Bioanalyser Chip (Agilent). The resulting libraries were sequenced at Genotypic Technology (Bangalore, India) using Illumina Hiseq platform.

The adaptor sequences used for the library preparation were Adaptor1:5′ AATGATACGGCGACCACCGAGATCTACACGTTCAGAGTTCTACAGTCCGA 3′ and Adaptor2: 5′CAAGCAGAAGACGGCATACGAGAT[index]GTGACTGGAGTTCCTTGGCACCCGAGAATTCCA 3′.

### Analysis of sRNAs

The raw reads of 150 bp from the libraries were first cleaned by removing 5' and 3' adaptors, low quality reads and reads with length < 16 or length > 36 nt (< 30 Q values) by using FASTAX-Toolkit. The remaining sequences from 16 nt to 36 nt long were used for further analysis. The clean reads were used for BLASTn search against Sanger RNA database (Rfam) (http://www.sanger.ac.uk/software/Rfam). Those reads which matched with other RNAs, like rRNA, tRNA, snRNA and snoRNA were excluded and the remaining reads were designated as filtered reads. These filtered reads were submitted to the miRProf pipeline (http://srna-workbench.cmp.uea.ac.uk/mirprof/) with default parameters which aligned the sequences to miRBase 16.0 (http://www.miRBase.org) and provided raw and normalized read counts of known miRNAs. miRBase 16.0 was used to identify the conserved miRNAs and the number of miRNA families in both the libraries. The miRNAs that were mapped on previously reported miRNAs were designated as conserved and unmapped as putative novel miRNAs. The novel miRNAs from the sRNA libraries were identified using the Mireap program (http://sourceforge.net/projects/mireap). The sequences were used to obtain all the candidate precursors with hairpinlike structures that were perfectly mapped by the sequencing tags. The secondary structures of putative pre-miRNA were checked using the Mireap structure creator with default parameters. Annotation of the whole data was carried out using the criteria described by Meyers et al. [13] where they have described various methods to annotate the plant miRNAs. The detailed protocol for the annotation of novel miRNAs has been explained in S1 File.

### Prediction of potential miRNA targets and their functional annotation

Target genes of miRNAs were predicted using the psRNA Target program (http://plantgrn.noble.org/psRNATarget). BLASTn hits with <3 mismatches were chosen as candidate targets, and afterward nucleotide 6-frame translation protein (blastx) was used to obtain the putative functions. In order to investigate the putative functions of potential target genes, the conserved miRNA target sequences were annotated using the Gene Ontology (GO) as well as Kyoto Encyclopedia of Genes and Genomes (KEGG) protein database. The Gene Ontology was categorized into three independent hierarchies namelymolecular functions, biological processes and cellular components. The enriched GO terms in our data in comparison to the total annotated miRNA genes were verified using Fisher’s test at a significant P-value of < 0.05. Also, target prediction for novel miRNAs was carried out using psRNATarget server.

### Determination of differential expression miRNAs

Differential expression of miRNAs in each library was obtained using log_2_-ratios and scatter plots. Initially, the miRNA expression levels were normalized to the expression of transcript per million (TPM). Subsequently, the fold-changes and p-values were calculated from the normalized expression values, which were further used to generate scatter plots and log_2_-ratios. Gene expression was considered to be significant when fold change was ≥ 1 and P value was ≤ 0.05. Heat maps showing differential expression profiles were generated using Multi Experiment Viewer (v4.8).

### Expression analysis of miRNAs using Real-Time (RT) PCR

During the present investigation, expression analysis was carried out for the miRNAs (both known and novel) targeting carotenoid, flavonoid and transcription factor genes (S2 File). miRNA specific stem-loop and RT primers (S2 File) were synthesized and RT-PCR was performed in both OR and PB carrot [14]. Further quantification of selected miRNAs was carried out using QuantStudio (TM) 6 Flex (Applied Biosystems, Foster city, USA) system. Tubulin was used as an internal control. For miRNA specific qRT-PCR, each 10 μl PCR reaction included 1 μl RT product, 10 mM specific forward primer, 10 mM universal reverse primer and 5 μl SYBR green PCR master mix. The reactions were incubated in a 96-well plate at 95°C for 10 min, followed by 40 cycles of 95°C for 15 s and 60°C for 1 min. Amplification specificity was verified using melt curve. Three technical replicates were taken and the size of the PCR products was checked on a 4% agarose gel. The expression data was normalized by using the expression values of tubulin and relative expression was calculated using 2^−ΔΔCt^ method. Fold change value was determined using 2^−ΔΔCt^ method [15]. Standard error was also calculated from the three technical replicates of the biological tissues. Students t test (P < 0.05) was conducted in order to check the significant differences in the expression of miRNA genes between OR and PB carrot.

### miRNA-Target validation using modified 5’ RLM-RACE approach

Modified 5’ RLM-RACE was performed in order to find out the cleavage site of the known and novel miRNAs on the target genes in both the color variants (PB and OR) using GeneRacer^TM^ kit (Invitrogen). Total RNA was ligated to the 5’ adaptor using T4 RNA ligase. From this RNA cDNA was synthesised using gene specific primers according to the protocols described by Roy et al. and Kaur et al. [6,16]. Further PCR reactions were carried out using 5’ adaptor specific forward primer and gene specific outer and nested inner reverse primers. The miRNAs, target genes and primers used for the RLM RACE have been given in S2 File. Phusion High-Fidelity DNA Polymerase (Thermo Scientific^TM^) was used for the amplification of the target genes using the PCR program provided with the kit with little modifications. The amplified PCR products were eluted and subjected to sequencing so as to map the cleavage site of miRNA on the target gene. Amplification and sequencing of each target gene was done in triplicates in order to confirm the cleavage site of the selected miRNAs.

## Results

### sRNA sequencing

**Fig 3:**
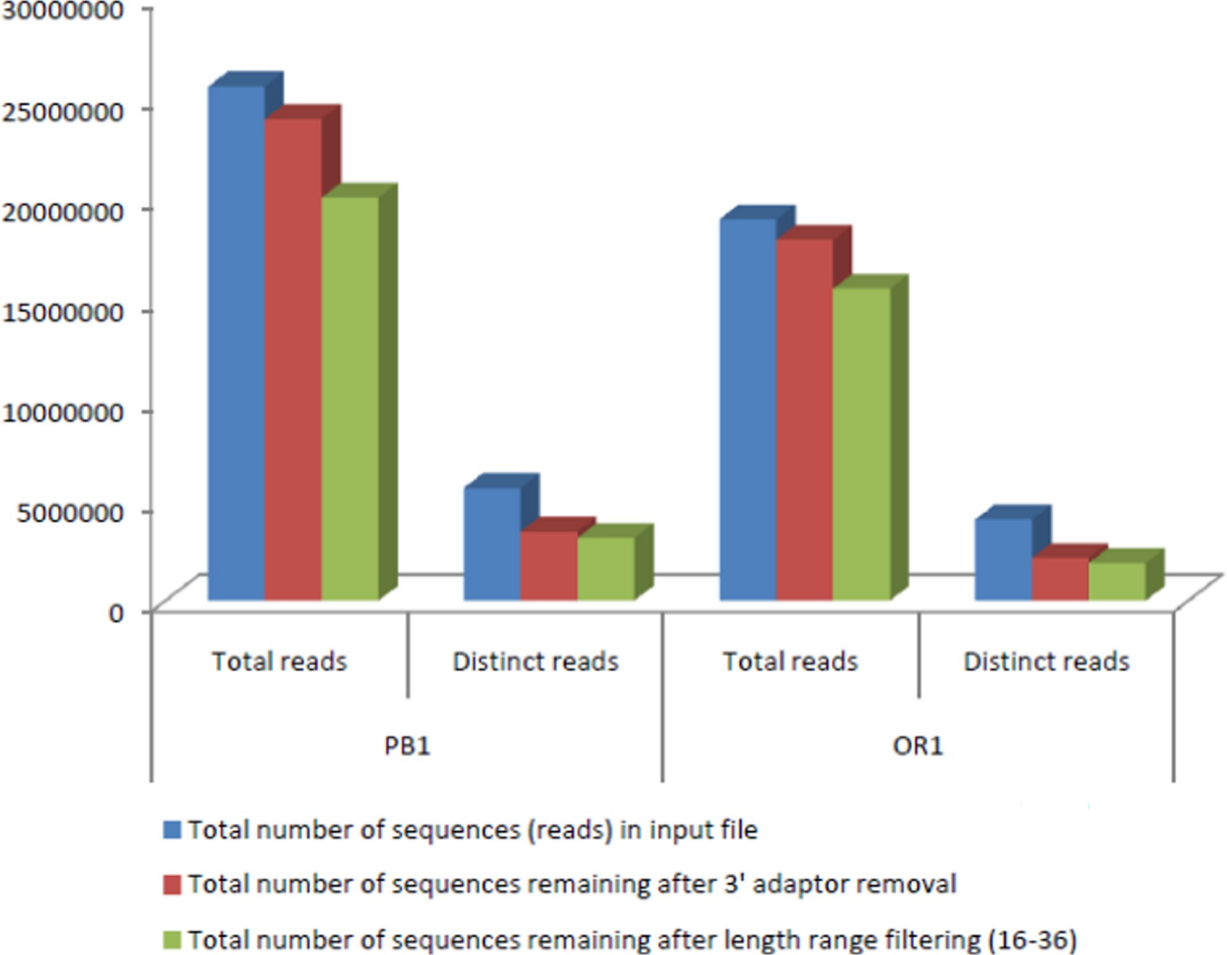
Total number of reads after sequencing.

The present study focused on genome-wide identification of miRNAs in two variants of carrot: Orange Red (OR) and Purple Black (PB), their expression profiles and possible regulatory implications particularly on the carotenoid and flavonoid pathways. The libraries were processed further for sequencing. In order to identify the candidate miRNAs in carrot, we sequenced small RNA libraries constructed from roots of PB and OR. A total of 25.5M and 18.9M reads were generated for PB and OR samples, respectively (Fig 3). After filtering the low quality reads, 3’ insert null, poly (A), length <16 or length >36, the majority of small RNAs were 21 to 25nt in length (S1 Fig). Length filtered reads were made unique reads by generating the read count (S2 Fig). The distribution of lengths of small RNAs was similar in the two libraries. All the sequences were checked for ncRNA (rRNA, tRNA, snRNA and snoRNA) contamination. The unaligned reads to ncRNAs were used for known miRNA prediction. Reads were made unique and the read count profile was generated, accordingly. The raw data of both the libraries has been submitted to NCBI database under accession numbers SRR6007613 and SRR6007614.

### Identification of known miRNAs and miRNA families

**Fig 4:**
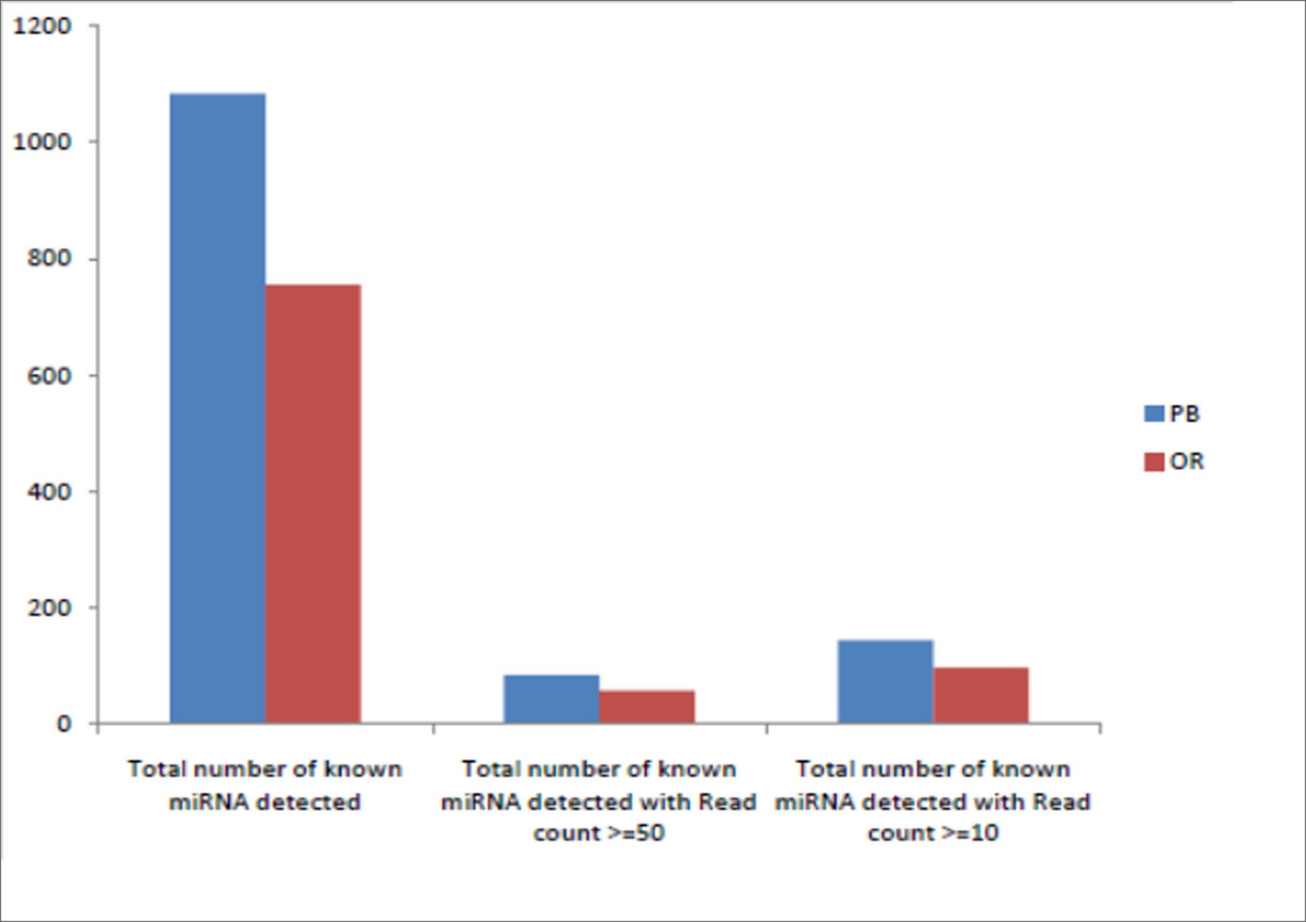
Total number of miRNAs in PB and OR variants.

In order to identify known miRNAs (both conserved and non-conserved), filtered reads were mapped with currently known mature plant miRNAs using miRBase 21.0. Homology search of the miRNAs was performed against Viridiplantae mature miRNA sequences retrieved from miRBase 21 database using NCBI-BLAST-2.2.30. In all 144 and 98 (read count >10) conserved miRNAs were identified from the PB and OR library, respectively (). A total of 430 known miRNA sequences belonging to 85 families were identified in the PB sample. Similarly, 288 known miRNAs belonging to 55 different families were identified in the OR sample. The family names were assigned based on the miRBase 21.0 data (S3 Fig). The number of miRNAs in each family varied from 2 to 32 in PB and 2 to 26 in OR (Fig 4). Most of the families comprised of 2–5 miRNAs; 19 families in OR and 31 in PB comprised at least five members. The average minimum free energy values in PB and OR were 23.58 and 24.83 kcal/mol, respectively.

### Identification of novel miRNAs

For the identification of novel miRNAs, sequences not showing hits with the known miRNAs were considered for prediction of novel miRNA using Mireap_0.2. MIREAP integrates miRNA biogenesis, sequencing depth and structural features to identify miRNAs and their expression level from deep sequenced small RNA libraries. *De novo* transcriptome of carrot wasused as a reference for the identification of miRNAs in both OR and PB carrot [17]. After searching for potential pre-miRNAs and predicting their hairpin-like structures, 36 and 66 unique sequences were identified as novel miRNAs in OR and PB varieties, respectively (S3 File). Three novel miRNAs, carrot-m0014, carrot-m0018 and carrot-m0030 were common in both the libraries. The novel miRNA sequences were 20-26nt in length. The range in the length of pre-miRNAs was 68 to 100nt in the PB with an average of 85nt and in the OR library, the length was 71-100nt with the average of 85 nt.

### Target prediction of novel miRNAs using psRNATarget

Novel miRNAs identified in the present study were subjected to psRNATarget to find out their respective target genes. Maximum number of miRNAs displayed cleavage mode of action and only a few of them showed translational inhibition (S4 File). Some of the targets included UDP-glycosyltransferase superfamily proteins, ethylene producing genes, transmembrane protein, calcium-binding proteins, kinase superfamily and many other carbon metabolic pathway genes. The cleavage was also observed for many important transcription factors like AGAMOUS, MADS-Box, Zinc finger, AP2, TCP family, GRAS, SBP, NAC, bHLH, bZIP, WD40, etc.

### Functional annotation of miRNA target genes

**Fig 5:**
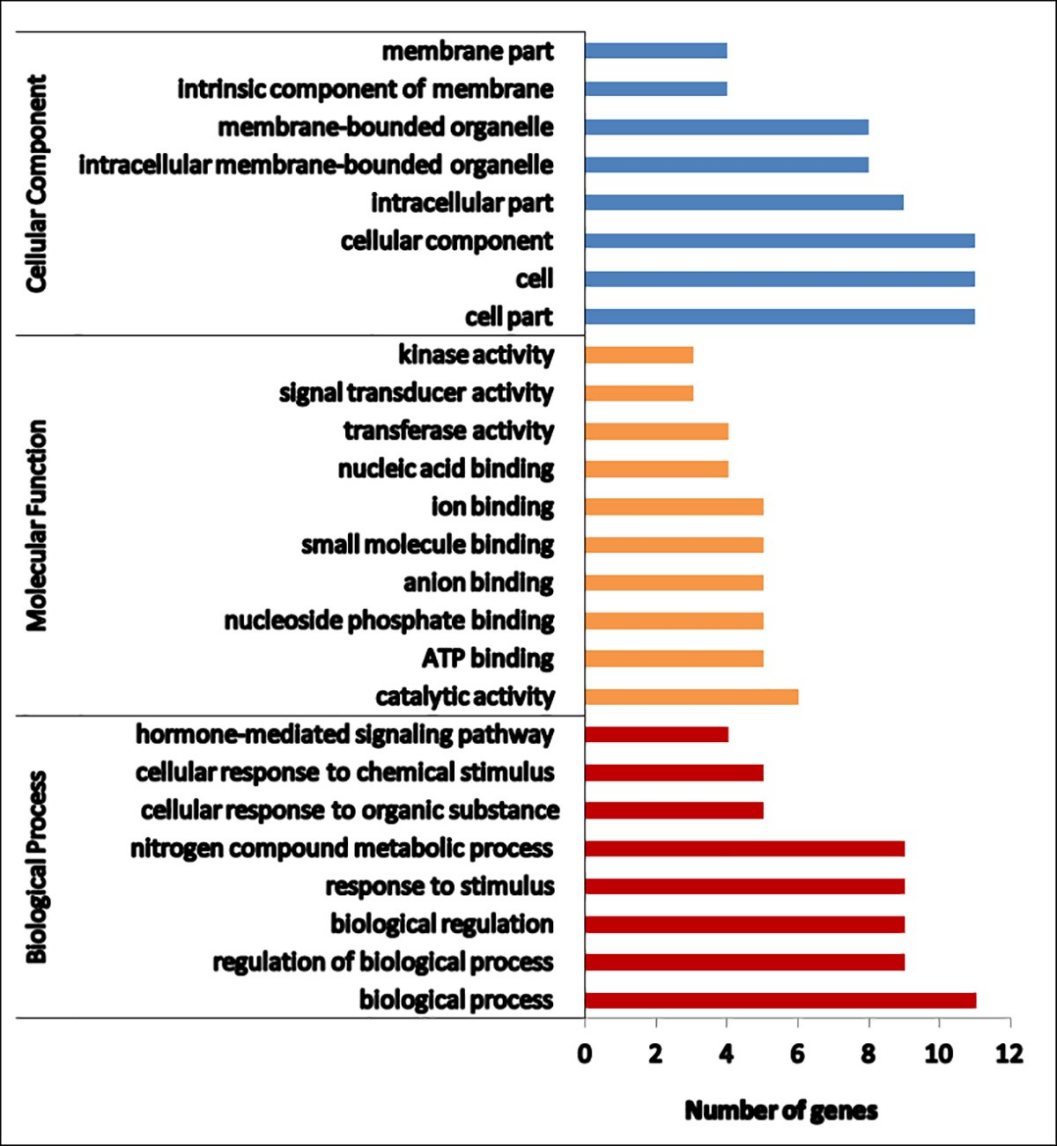
Functional annotation of predicted targets of miRNAs in OR carrot. Graph is showing various processes under different functional categories.

**Fig 6:**
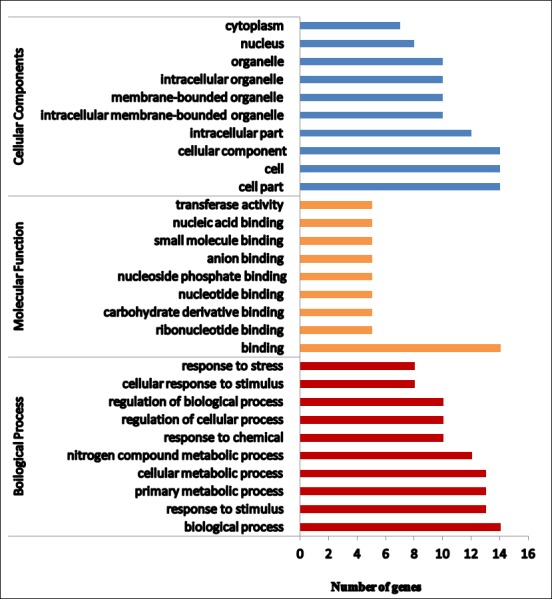
Functional annotation of predicted targets of miRNAs in PB carrot. Graph is showing various processes under different functional categories.

For understanding the functions of the identified miRNAs, putative targets were predicted using miRanda-3.3. Novel miRNAs and known miRNAs with copy number > = 10 were considered for target prediction. A total of 714 and 1082 potential target genes were identified in OR and PB, respectively. GO analysis and KEGG annotations were performed to evaluate the potential functions of these miRNA target genes (S5 File). On this basis, the miRNA target genes were categorized according to molecular function, biological processes and cellular components. In OR, out of 714 targets, 461 belonged to the molecular functions, 287 to the biological processes and 333 to the cellular components (Fig 5). In PB, 780 belonged to the molecular functions, 449 to the biological processes and 563 to the cellular components (Fig 6). The molecular functions were further classified into ten categories, among which the most over-represented GO terms were ATP binding followed by DNA binding. Ten different biological processes were observed, the most frequent term being the integral component of membrane. In the cellular component, the nucleus represented the majority portion. KEGG pathway analysis was performed to examine the biological interpretation of these target genes. Forty eight different pathways were found in PB and the most represented pathways included those for RNA transport (eleven enzymes), plant hormone signal transduction (ten enzymes), glycolysis/ gluconeogenesis (nine enzymes), starch and sucrose metabolism (nine enzymes and endocytosis (eight enzymes). In OR, forty different pathways were found and the more profound were plant hormone signal transduction, RNA degradation, cysteine and methionine metabolism, glycolysis and mRNA surveillance (six enzymes each).

### Expression pattern of miRNAs in PB and OR

Next, we carried out DGE analysis using DESeq tool so as to gain insights into the putative roles of miRNAs. An absolute threshold value of the log_2_ ratio fold change >1 was used to determine the statistical significance of the miRNA abundance. A total of 472 differentially expressed miRNAs were selected from the two libraries. Scatter plot showing differential expression of miRNAs between OR and PB has been shown in “S4 Fig”. 130 miRNAs were up regulated and 47 were down regulated, while the expression of 295 miRNAs was found to be neutral. Heat maps were generated for each set of DGE using top miRNAs (up/down regulated) (S5 Fig). Based on the abundance, some families of miRNAs exhibited considerable differences, between the two varieties. For example, csi-miR166a and gma-miR6300 were more abundant in OR variety, whereas ppt-miR390b and zma-miR 396g-3p were more abundant in PB carrot. Most of the miRNAs, which showed huge differences in expression between the varieties, were found to be involved in multiple pathways. The expression of gma-miR171k-3p was calculated to be 193.75 times more in PB than OR. The functional annotations of the gene revealed its involvement in thirteen different pathways. Similarly, another differentially expressed miRNA, mdm-miR167i, was found to be involved in sixteen different pathways.

### Identification and enrichment analysis of miRNAs target genes

As carrot represents a model plant showing variation in the development of color in root tissues, miRNAs targeting important carotenoid and anthocyanin genes were predicted using sRNA target prediction tool; psRNATarget with default parameters. In OR library, 27 miRNAs targeted the carotenoid and flavonoid pathways genes, whereas in PB library, 39 targeted these pathway genes. To further gain insight into the regulation of these pathways by miRNAs, heat maps were generated.

**Fig 7:**
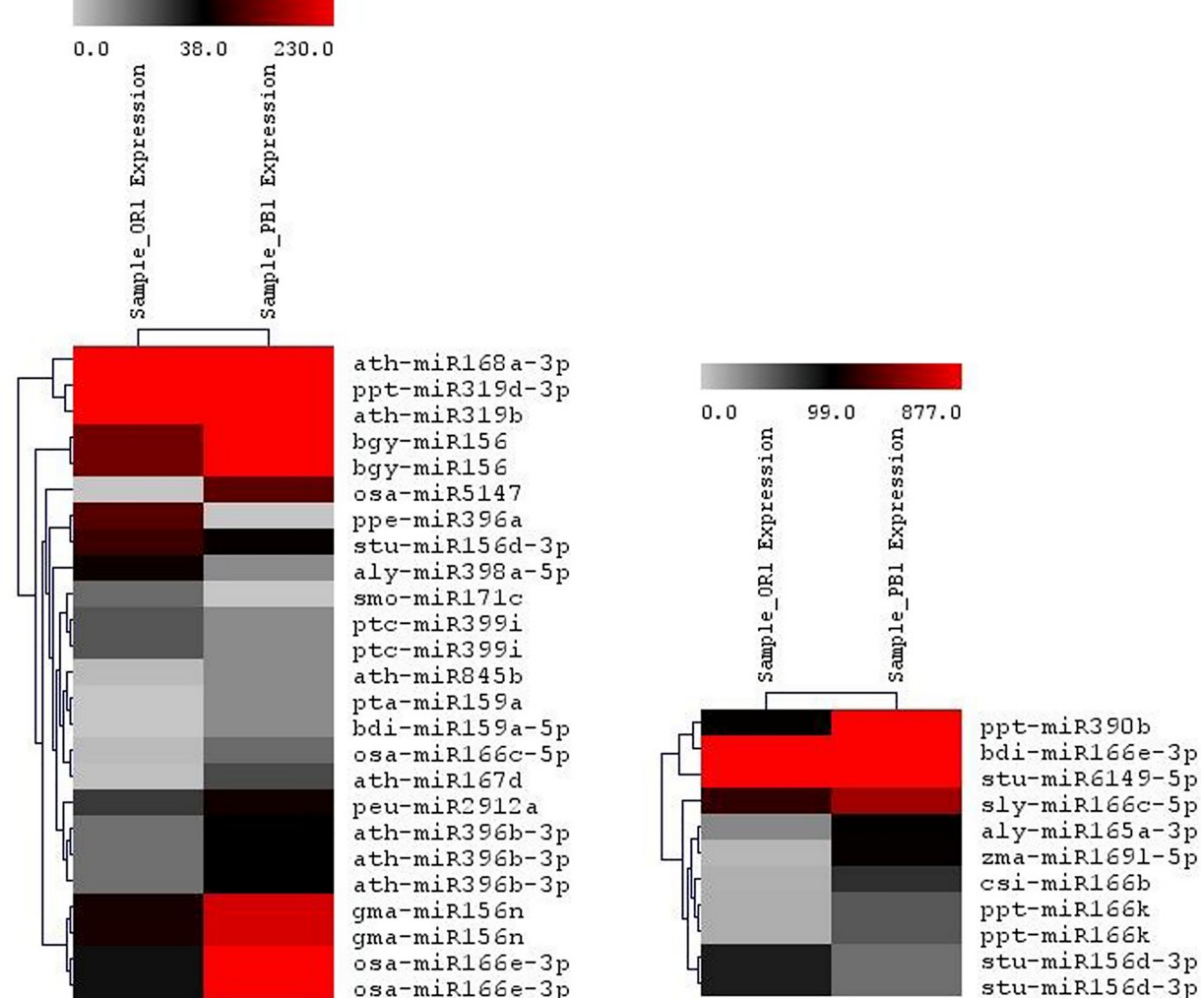
**Heat map showing differential expression of miRNAs regulating a) carotenoid and b) flavonoid pathway in OR and PB**.

In the present study, we found the miRNAs targeting both the carotenoid as well as flavonoid pathway genes. The differential expression patterns of the miRNAs targeting the carotenoid biosynthesis revealed that most of the genes were upregulated in PB variety as compared to OR (Fig 7). For instance, six miRNAs namely, ppt-miR319d-3p, pta-miR159a, gma-miR156n, bgy-miR156, gma-miR156n and bgy-miR156 were found to target phytoene synthase (*psy*) gene of the carotenoid biosynthetic pathway. The expression of all these six miRNAs was found to be upregulated in the PB variety by >4-fold as compared to the OR variety. Similarly, ath-miR396b-3p targeting zeta-carotene desaturase (*ZDS*) was found to be upregulated in the PB by almost 2-fold as compared to the OR variant. In case of flavonoid pathway, the expression of most of the miRNAs was found to be neutral between the two varieties, whereas a few miRNAs were found to be upregulated in the PB variety (Fig 7).

### Validation of miRNA expression by qRT-PCR

**Fig 8:**
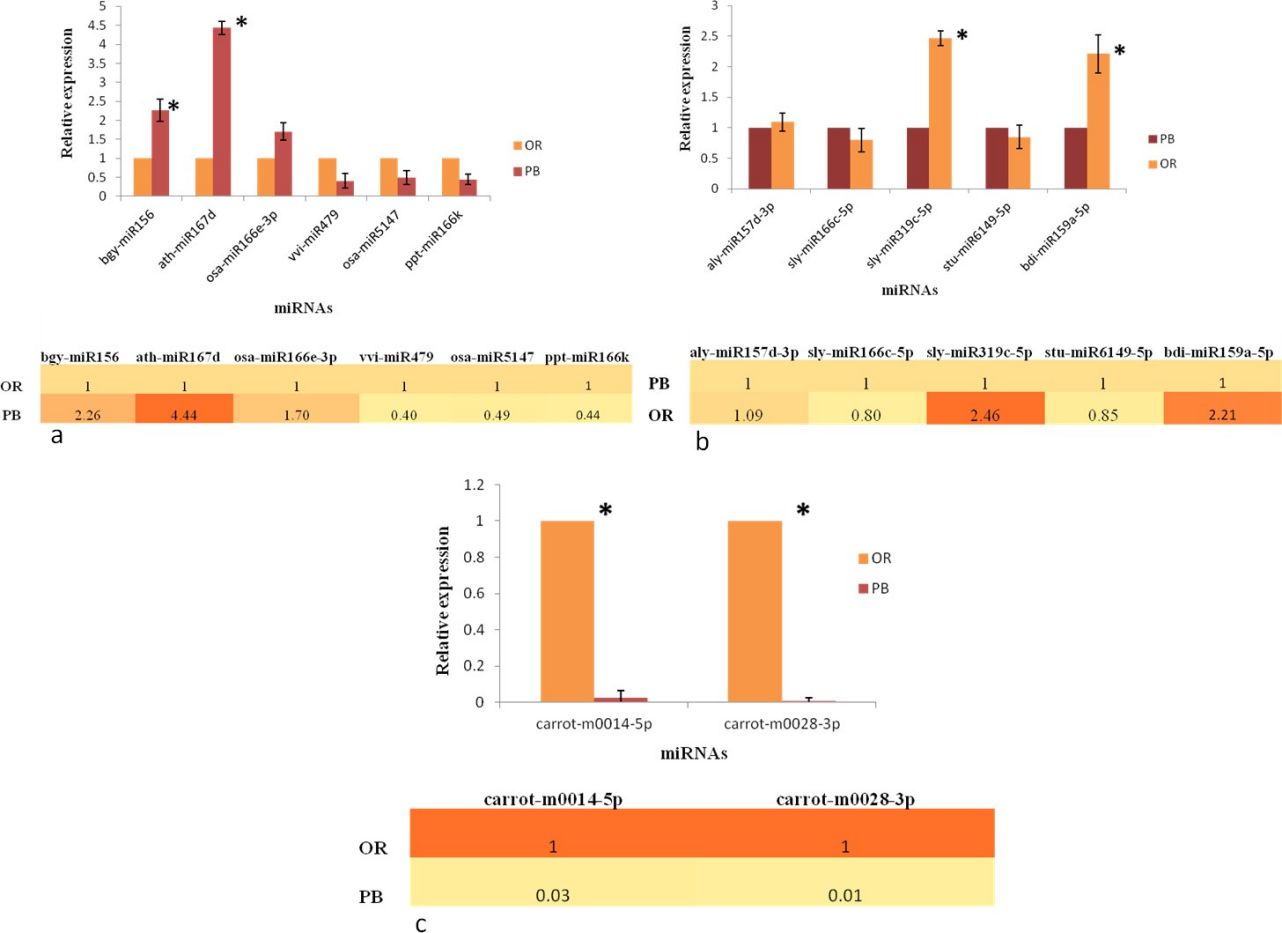
**Transcript profiles of the miRNAs targeting a) carotenoid genes b) flavonoid genes and c) Transcription factor genes using qRT-PCR.** For normalizing the gene expression level, tubulin was selected as an internal control. All experiments were conducted using the three technical replicates of the biological tissues. All the fold changes were calculated using ΔΔCt method that shows the change in gene expression level in the OR and PB carrot. Standard error was calculated from the average of three technical replicates of the biological tissues. Student’s t-test (P < 0.05) was performed in order to determine the significant differences in the miRNA expression levels between OR and PB sample. The significant differences (P < 0.05) obtained has been marked as * in the figure.

Out of differentially expressed miRNA families predicted by deep-sequencing, a total of 11and 2 novel miRNAs targeting important carotenoid, flavonoid and transcription factor genes were chosen for qRT-PCR analysis (S2 File). qPCR of 13 miRNA candidates exhibited differential expression in PB and OR carrot (Fig 8). miRNAs targeting carotenoid genes were up-regulated in PB carrot as compared to OR e.g. bgy-miR156 targeting phytoene synthase gene showed one fold higher expression in PB than its counterpart. Similarly, ath-miR319b targeting lycopene-beta cyclase showed 3 fold higher expression in PB as compared to OR. On the other hand, miRNAs vvi-miR479 and osa-miR5147 displayed slightly higher expression in OR as compared to PB carrot. As far as miRNAs targeting flavonoid pathway genes are concerned, they showed almost neutral expression in both the varieties, except sly-miR319c-5p which targets shikimate O-hydroxycinnamoyltransferase, showed one fold higher expression in OR. Novel miRNAs i.e. carrot-m0014-5p and carrot-m0028-3p, targeting transcription factor genes (TCP and AP2) displayed higher expression in OR as compared to PB variant (Fig 8).

### Validation of miRNA target genes

**Fig 9:**
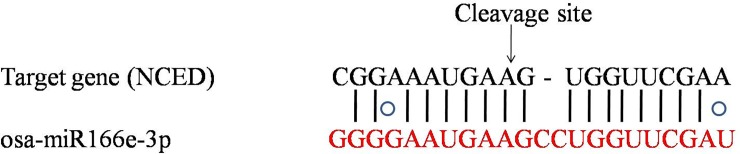
Cleavage site detection using RLM-RACE approach. 5' RLM RACE was employed so as to map the cleavage sites. The coding sequence of the target gene was aligned with the miRNA and the arrow above the aligned sequences indicates the cleavage site.

During the present study, targets of 3 conserved miRNAs and 2 novel miRNAs were validated through 5' RLM-RACE approach in both PB and OR carrot (S2 File). However, cleavage was only found for 1 target gene i.e. 9-cis-epoxycarotenoid dioxygenase (NCED). The cleavage site of NCED was detected at 11^th^ position from 5’ end of the miRNA sequence (Fig 9).

### Statistical data analysis

All the present experiments were carried out twice taking three biological replicates. Standard mean of three replicates was calculated along with the standard deviation which is shown as the error bars in the experimental graphs. A value of P < 0.05 (t-test) suggested that variations obtained during the present study are statistically significant.

## Discussion

Carrot has the rare distinction of storing carotenoids and anthocyanins in roots. These metabolites are synthesized by following two distinct metabolic pathways. In plants, most of the studies on both these metabolic pathways have been on the above ground parts; however, much remains to be explored about roots. Therefore, carrot represents an ideal system to understand the mechanism of carotenogenesis and biosynthesis of flavonoids in roots.

miRNAs are a group of small non-coding RNAs that play important roles in various developmental and stress-responsive processes by regulating the gene expression [18]. Many studies have focused on miRNA profiling in various plants. However, to the best of our knowledge, only seventeen miRNAs have been identified in carrot, using *in silico* analysis [19] while no experimental validation has been performed for identifying and analyzing miRNAs. The present study focused at genome-wide discovery of miRNAs in two varieties of carrot having contrasting phenotypes i.e. PB and OR, their expression profiles and possible regulatory implications, particularly on the carotenoid and flavonoid pathways.

High throughput sequencing technology has been extensively applied in the small RNA research. Thousands of miRNAs along with their functions have already been identified in many plants. Regarding carrot, a single report regarding transcriptome has been published till date and hence comprehensive miRNA information is not available [17]. During the present study, RNA-Seq was employed to understand the differential gene expression of miRNAs vis-à-vis color development. To identify the candidate miRNAs, we sequenced small RNA libraries constructed from roots of the two varieties. Illumina sequencing resulted in the generation of 25.5M and 18.9M reads in PB and OR libraries, respectively.

The size distribution of the filtered sequence reads indicated the high quality of data (S2 Fig). The largest fraction of small RNAs was 24nt long, indicating the abundant representation of endogenous siRNAs. Overall more than 80% small RNAs were within the range of 21-25nt as expected. The above observations are consistent with DCL cleavage products and those reported in previous studies [20,21].

sRNA sequences were compared with currently known mature plant miRNAs using miRBase 21.0, in order to identify known miRNAs in the two carrot varieties. Most miRNAs of different lengths harbored a uridine residue at the 5’ end i.e. the majority of miRNAs tended to start with 5’U. Similar, size distribution and prevalence of 5’U has been observed for known miRNAs in other plant species [21,22]. For identification of novel miRNAs, sequences not showing hits with the known miRNAs were considered for prediction of novel miRNA using Mireap_0.2. The average minimum free energy values in ‘PB’ and ‘OR’ were 23.58 and 24.83 kcal/mol, respectively which are in consonance with the -23.18 and -31.12 kcal/mol in celery and much higher than other plant pre-miRNAs (-59.5 and -71.0 kcal/mol in *Arabidopsis* and rice, respectively) [23]. This shows that the microRNA precursors have lower folding free energies, unlike other non-coding RNAs sequences. The length of miRNAs and nucleotide preference distributions are shown in S3 Fig. The 21-nt-long miRNA with 5′-uridine is a characteristic feature of DCL1 cleavage and AGO1 association, which has been found in most known miRNAs [24,25].

Several sequences belonging to a large number of miRNA families were identified in both PB and OR samples. Such large gene families have been reported in other plants as well [21,26]. Most of the miRNAs, which were identified during the present investigation showed high sequence similarities to miRNAs in other plants. The distribution of all the miRNA families was similar between the libraries. Most of the miRNA families were less abundant whereas some of the families such as miR166, miR159, miR482, miR171_1, miR156, miR396, miR164 and miR167 were abundant in the libraries. Similar results were obtained in chickpea, where the differential abundance of both conserved as well as novel miRNAs was observed [21].

The differences between the miRNA profiles of the two carrot varieties are possibly related to their different phenotypes and climate. The differential abundance, as observed for the 472 miRNAs between the OR and PB, was significant (S4 Fig). Different miRNA genes accounted for a large proportion of all the identified miRNAs. These findings can be explained on the basis of the fact that the two carrot varieties show different phenotypes and are supposed to have different geographical origin. From the present study, it appears that miRNA evolution in the same plant species is more conservative and that variations in the miRNA profiles of the two carrot varieties may be critical for the differences in the miRNA expression. The characterization and comparative profiling of entire sets of small RNA (small RNA transcriptome), especially the miRNA transcriptome, has laid the foundation to unravel the complex miRNA mediated regulatory networks controlling carotenoid and flavonoid pathway gene expression in PB and OR carrot.

We examined the miRNAs involved in the color development pathways which were differentially expressed between the two variants. The expression of the miRNAs targeting carotenoid genes was 1–2 folds higher in PB carrot as compared to OR carrot. The higher level of this expression in PB might downregulate the early steps of carotenoid biosynthesis pathway and hence minimize the carotenoid biosynthesis as compared to the anthocyanins in the roots. Xu et al. [27] carried out some studies on miRNAs in sweet orange where they found 418 potential target genes, out of which two targets included important carotenoid biosynthetic pathway genes i.e. *ggpps* targeted by csi-mir167 and *lcy-b* targeted by csi-miR1857. Similarly, expression of miRNAs targeting anthocyanin pathway was almost neutral in both the variants except some which showed slightly higher expression in PB carrot. This can be attributed to the reason that miRNAs do not always negatively correlate with their targets [28,29].

miRNAs targeting carotenoid genes showed higher expression in PB as compared to OR. Therefore, it can be expected that the higher expression of miRNAs leads to the minimum expression of carotenoid genes in PB, which is clear from the phenotype. Slightly higher expression of vvi-miR479 targeting carotenoid isomerase (*crtISO*) gene in OR can be attributed to the fact that miRNAs can interact positively with the target genes. Earlier, we have reported positive regulation of *crtISO* gene by miR172, at different developmental stages of tomato [29]. Similar findings were made in case of the flavonoid pathway, where expression of miRNAs was almost neutral in PB and OR except sly-miR319c-5p which might be positively regulating the target flavonoid gene i.e. shikimate O-hydroxycinnamoyltransferase.

In plants, color development is known to be regulated by the interconnected mechanisms like transcription factors and their interactions with miRNAs, other regulatory factors and is not an act of straight regulation by a single or a few miRNAs [30]. Therefore, it is difficult to elucidate the molecular mechanism behind the miRNA-mediated regulation of color development in plants. From the past decade, miRNAs have been identified as a part of the regulatory mechanisms in all organisms. These can be useful tools for altering the accumulation of carotenoids and flavonoids in the crop plants (for edible parts) and in ornamental plants. Tissue -specific use of miRNAs can prove to be an ideal tool for the crop improvement and production. The present study represents the first report on the identification and investigation of miRNAs in carrot vis-à-vis color development. Further, deep-sequencing of miRNA of these two contrasting carrot variants will certainly provide a potential clue about the color development in plants.

## Conclusions

One of the essential aspects of small RNA regulated processes includes the elucidation of various mechanisms involved in color development. In the present study, differential gene expression of miRNAs was reported in two color variants of carrot. A total of 472 differentially expressed miRNAs were observed in Orange Red and Purple black carrot. Of these, 130 miRNAs were up regulated, 47 down regulated and 295 neutrally expressed. In terms of abundance, huge differences were observed among some miRNA families in the two varieties. 27 miRNAs in OR variety targeted both the carotenoid and flavonoid pathway, whereas the number in PB was 39. Most of the miRNAs in the carotenoid biosynthesis were upregulated in PB variety, which supports the high expression of carotenoid pathway genes in OR as compared to PB. Therefore, it is expected that these miRNAs might be playing a critical role in cultivar-specific color development processes. The combined miRNA and their target gene analysis suggested that differential expression of miRNAs in two color variants of carrot might be involved in the regulation of pigment biosynthesis and accumulation. Further, *in-silico* characterized and validated novel miRNAs might provide an important clue to regulate the color development in vegetable crops.

## Supporting information

S1 Fig First nucleotide bias of miRNAs in (a) Orange Red (b) Purple Black.
(TIF)

S2 Fig Small RNA length distribution of unique reads. (TIF)

S3 Fig Identical miRNA members in each family in Orange Red and Purple Black. (TIF)

S4 Fig Scatter plot showing differential expression of miRNAs between Orange Red and Purple Black. (TIF)

S5 Fig Heatmap showing tissue-specific expression of miRNAs. The scale represents log_2_ transformed normalized expression.
(TIF)

S1 File Detailed protocol for the annotation of novel miRNAs. (DOCX)

S2 File List of primers used for Stem-loop, qRT-PCR and RLM-RACE. (XLSX)

S3 File Novel miRNA candidates in OR and PB Variant. (XLSX)

S4 File *In silico* identification of the novel miRNA target genes in PB and OR carrot. (XLS)

S5 File Gene Ontology (GO) of miRNA targets in OR and PB. (XLSX)
