# Supplementary figures and images for: Identification and expression profiling of miRNAs in two color variants of carrot (Daucus carota L.) using deep sequencing
Source: PLoS One. 2019 Mar 7;14(3):e0212746. doi: 10.1371/journal.pone.0212746 (PMC6405255; doi:10.1371/journal.pone.0212746)

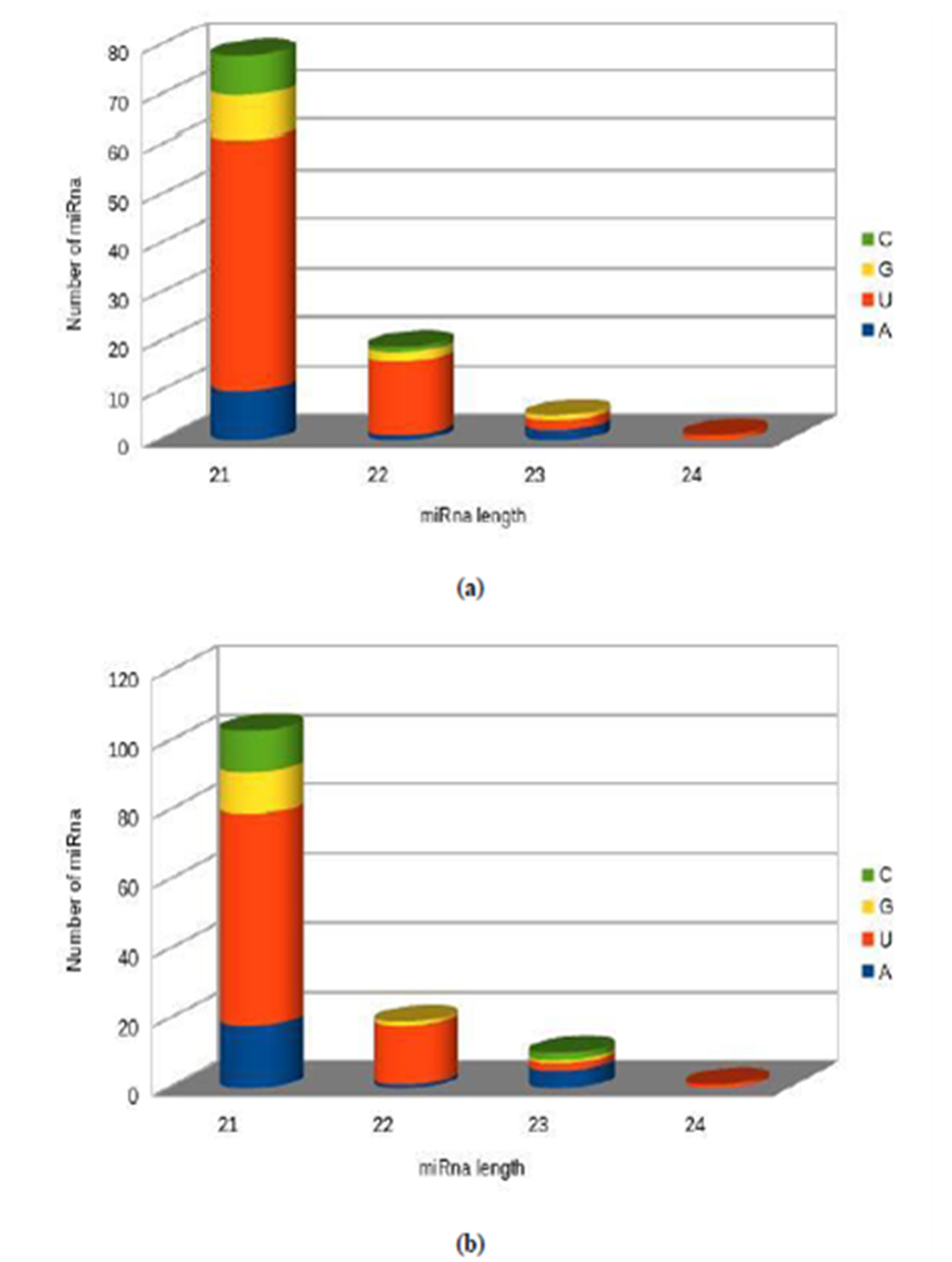

Supplement: S1 Fig — First nucleotide bias of miRNAs in (a) Orange Red (b) Purple Black. (TIF) [file pone.0212746.s001.tif]

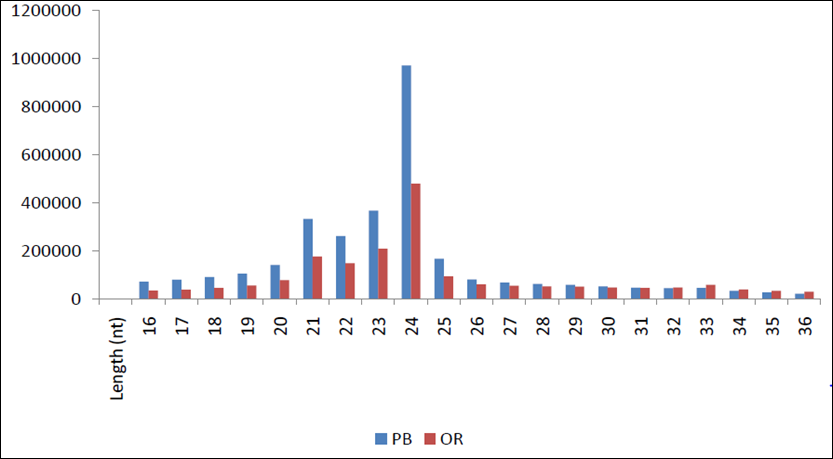

Supplement: S2 Fig — (TIF) [file pone.0212746.s002.tif]

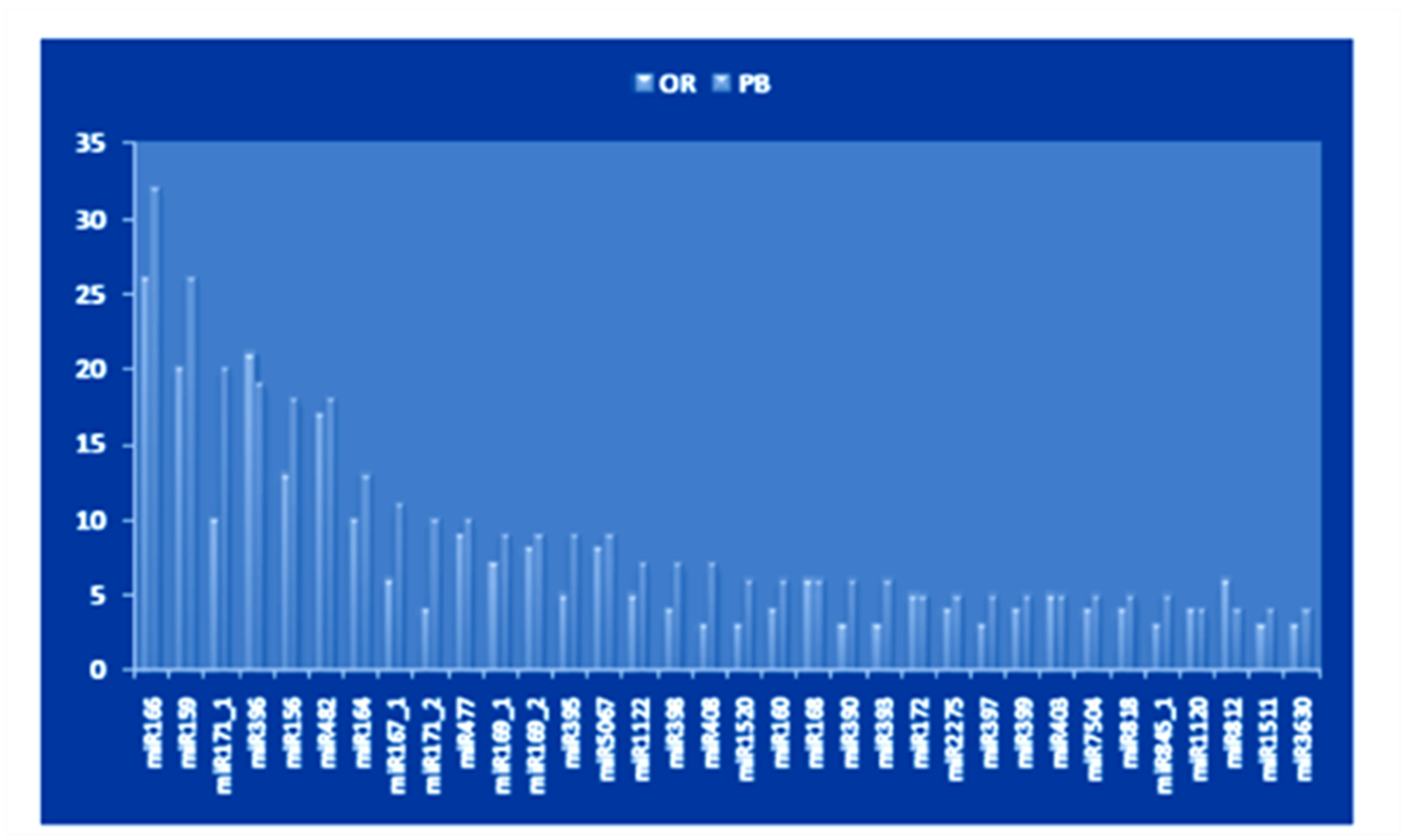

Supplement: S3 Fig — (TIF) [file pone.0212746.s003.tif]

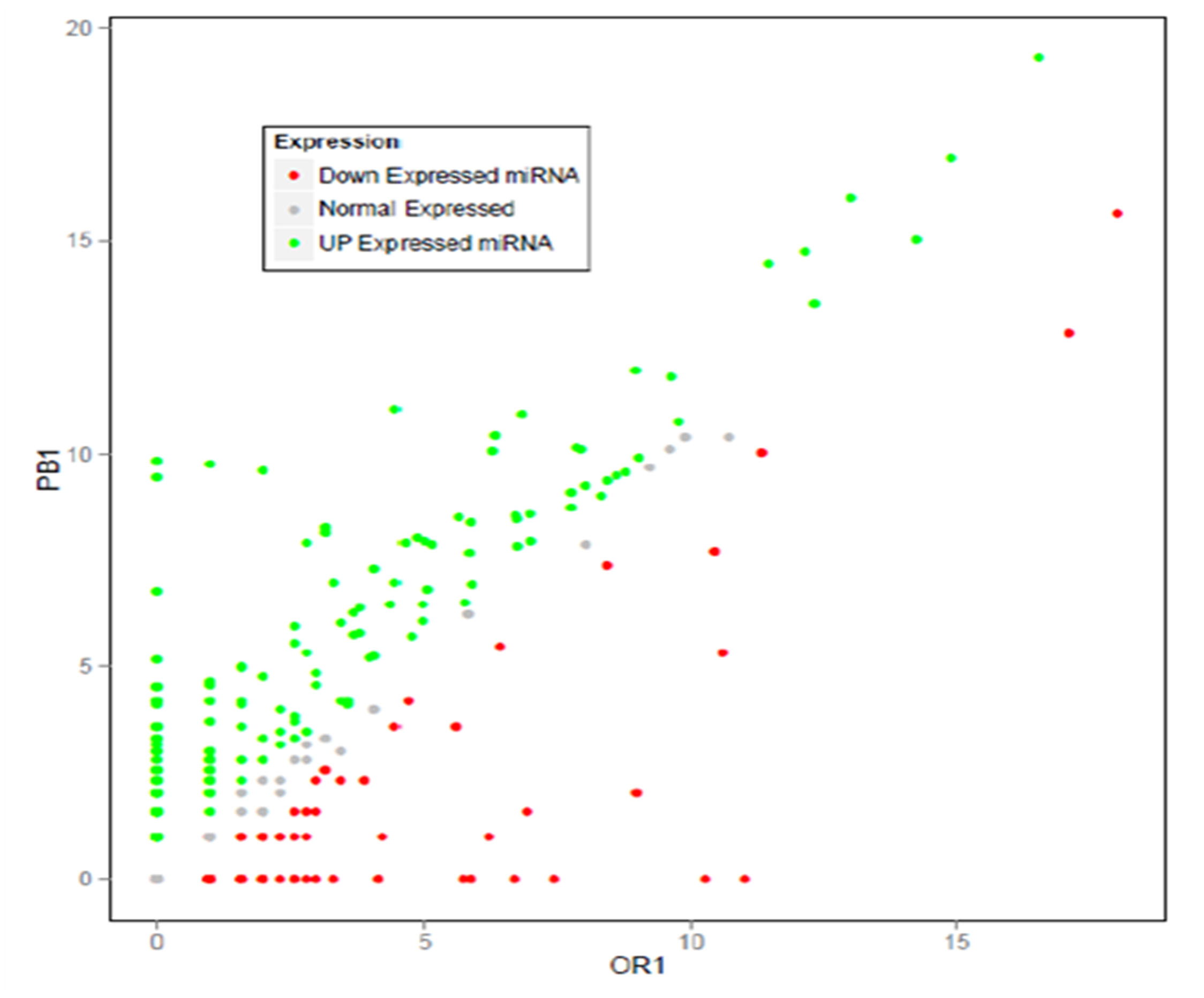

Supplement: S4 Fig — (TIF) [file pone.0212746.s004.tif]

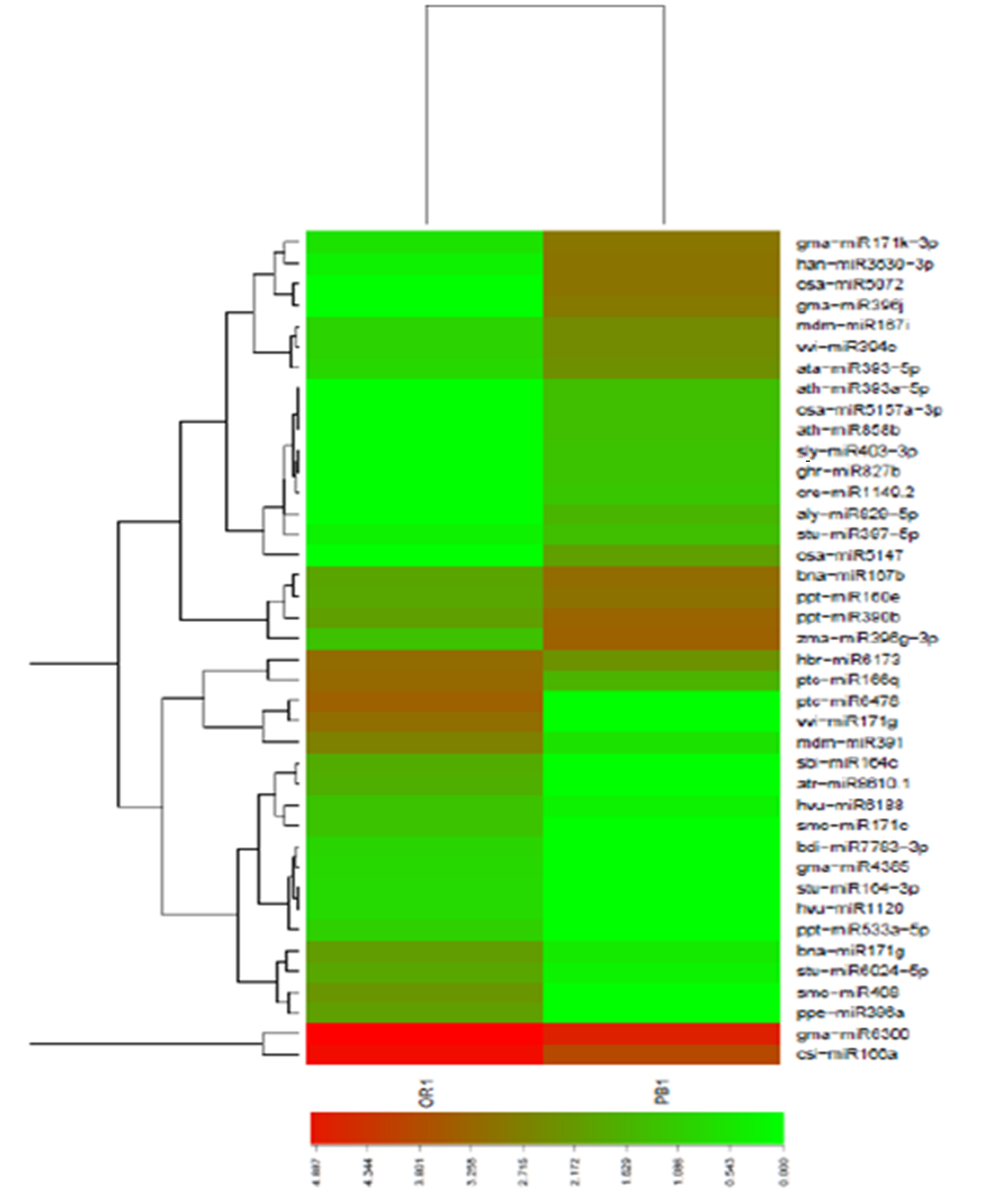

Supplement: S5 Fig — The scale represents log2 transformed normalized expression. (TIF) [file pone.0212746.s005.tif]
