# Supplementary material for: Identification and expression profiling of miRNAs in two color variants of carrot (Daucus carota L.) using deep sequencing
Source: PLoS One. 2019 Mar 7;14(3):e0212746. doi: 10.1371/journal.pone.0212746 (PMC6405255; doi:10.1371/journal.pone.0212746)
Supplement: S1 File — (DOCX) [file pone.0212746.s006.docx]

**Supplementary Information**

**Identification and expression profiling of miRNAs in two color variants of carrot (*Daucus carota* L.) using deep sequencing**

**Bhavana Bhan, Archana Koul, Deepak Sharma , Malik Muzafar Manzoor, Sanjana Kaul, Suphla Gupta and Manoj K. Dhar***

Correspondence should be addressed to M.K.D. (Email: [manojkdhar@rediffmail.com](mailto:manojkdhar@rediffmail.com))

**Detailed protocol for the annotation of novel miRNAs**

**Method**

**Identification of Novel miRNAs**

The remaining sequences were taken as input to identify novel miRNAs using the Mireap program (http://sourceforge.net/projects/mireap). These sequences were used to obtain all the candidate precursors with hairpin like structures that were perfectly mapped by sequencing tags. The secondary structures of putative pre-miRNA were checked using the Mireap structure creator. The parameters of Mireap 0.2 used were as follows: (i) minimum length of miRNA sequence is 18 nt, (ii) maximum length of miRNA sequence is 26 nt, (iii) minimum length of miRNA reference sequence is 20 nt, (iv) maximum length of miRNA reference sequence is 24 nt, (v) maximum copy number of miRNAs in the reference genome is 20, (vi) MFE for a miRNA precursor is -18 kcal/mol (vii) maximum distance between miRNA and miRNA is 300 nt, (viii) minimum length in the pairs of miRNA and miRNA is 19 bp, (ix) maximum bulge of miRNA and miRNA is 8, (x) maximum asymmetry of miRNA/miRNA duplex is 4 and (xi) flank sequence length of miRNA precursor is 20 nt. RNA sequences were considered miRNA candidates only if they satisfied the following criteria (Meyers *et al*., 2008), such as (i) formation of appropriate stem-loop hairpin secondary structure, (ii) location of mature miRNA sequence in one arm of the hairpin structure, (iii) less than 2 nt mismatches of miRNAs with the opposite miRNA sequence in the other arm, (iv) miRNA sequences without any loop or break and (v) prediction of secondary structures with higher MFEIs, negative MFEs, and 30–70% A+U contents.

**Result**

**Identification of novel miRNAs**

For identification of novel miRNAs, sequences not showing hits with the known miRNAs were considered for prediction of novel miRNA using Mireap_0.2. MIREAP integrates miRNA biogenesis, sequencing depth and structural features to identify miRNAs and their expression level from deep sequenced small RNA libraries. Stemloop hairpins were retained only when they complied with two requirements namely, i) the mature miRNAs-associated reads mapped in the arm region of the precursors, and ii) the free energy of the secondary structure calculated by RNA fold is lower than -18kcal/ mol. Carrot transcriptome sequences were used as reference. After searching for potential pre-miRNAs and predicting their hairpin like structures, 36 and 66 unique sequences were identified as novel miRNAs in Orange Red and Purple Black varieties, respectively. Three novel miRNAs, carrot-m0014, carrot-m0018 and carrot-m0030 were common in both the carrot varieties. The novel miRNA sequences were 20-26nt in length. The range in the length of pre-miRNAs was 68 to 100nt in Purple Black with an average of 85nt and in the Orange Red variety the length was 71-100nt with the average of 85. The average minimum free energy values in ‘Purple Black’ and ‘Orange Red’ were 23.58 and 24.83 kcal/mol, respectively which are in consonance with the -23.18 and -31.12 kcal/mol in celery and much higher than other plant pre-miRNAs (-59.5 and -71.0 kcal/mol in *Arabidopsis* and rice, respectively). Most miRNAs of different lengths had a uridine residue at the 5′ end. Other plant species are also known to have the prevalence of 5′ uridine. The 21-nt-long miRNA with 5′-uridine is a characteristic feature of DCL1 cleavage and AGO1 association, which has been found in most known miRNAs.
